# Comparison of Midazolam and Diazepam for Sedation in Patients Undergoing Double-Balloon Endoscopic Retrograde Cholangiopancreatography: A Propensity Score-Matched Analysis

**DOI:** 10.3390/jcm14072287

**Published:** 2025-03-27

**Authors:** Yuki Fujii, Kazuyuki Matsumoto, Akihiro Matsumi, Kazuya Miyamoto, Daisuke Uchida, Shigeru Horiguchi, Koichiro Tsutsumi, Toshiharu Mitsuhashi, Motoyuki Otsuka

**Affiliations:** 1 Department of Gastroenterology and Hepatology, Okayama University Graduate School of Medicine, Dentistry and Pharmaceutical Science, Okayama 700-8558, Japan; pmug1j9r@s.okayama-u.ac.jp (Y.F.); pxbb7o3o@okayama-u.ac.jp (A.M.); p5vn01ag@okayama-u.ac.jp (K.M.); pt77172s@s.okayama-u.ac.jp (D.U.); p4nc20ad@okayama-u.ac.jp (S.H.); tsutsumi@cc.okayama-u.ac.jp (K.T.); otsukamoto@okayama-u.ac.jp (M.O.); 2 Center for Innovative Clinical Medicine, Okayama University Hospital, Okayama 700-8558, Japan; sankyoh@gmail.com

**Keywords:** adverse events, balloon-assisted ERCP, benzodiazepine, sedation

## Abstract

**Objective:** The sedation method used in double-balloon endoscopic retrograde cholangiopancreatography (DB-ERCP) varies across countries and between healthcare facilities. No previous studies have compared the effects of different benzodiazepines on sedation during endoscopic procedures. This study aimed to compare the effects of midazolam and diazepam sedation on DB-ERCP outcomes. **Methods:** This retrospective cohort study analyzed consecutive patients who underwent DB-ERCP between January 2017 and February 2024. A total of 203 patients who were sedated with diazepam (*n* = 94) or midazolam (*n* = 109) were analyzed. Propensity score matching was applied to adjust for baseline group differences. The primary outcome was the incidence of sedation-related adverse events (AEs). Secondary outcomes included inadequate sedation requiring additional sedatives and risk factors for sedation-related AEs. **Results:** Sedation-related AEs were more frequent with diazepam (28% [21/75]) than with midazolam (14% [11/75]; *p* = 0.046). Hypoxia occurred more frequently with diazepam (19% [14/75]) than with midazolam (5% [4/75]; *p* = 0.012). However, no significant differences were observed between the two groups for hypotension (*p* = 0.41) and bradycardia (*p* = 1.0). Poor sedation requiring other sedatives occurred significantly more often with diazepam (8% [6/75]) compared with midazolam sedation (0% [0/75], *p* = 0.012). Multivariate analysis identified diazepam sedation (odds ratio, 2.3; 95% confidence interval, 1.0–5.3; *p* = 0.048) as the sole risk factor for sedation-related AEs. **Conclusions:** Midazolam is safer and more effective than diazepam sedation in patients undergoing DB-ERCP.

## 1. Introduction

The endoscopic treatment of biliary and pancreatic diseases in patients with postoperative intestinal reconstruction has traditionally been challenging because of the difficulty in accessing the papilla or hepaticojejunostomy/pancreatojejunostomy (HJ)/pancreatojejunostomy (PJ) using conventional endoscopes [1]. The development of balloon-assisted endoscopy (BAE) has enabled access to the biliary and pancreatic ducts in these patients [2]. Double-balloon endoscopic retrograde cholangiopancreatography (DB-ERCP) requires longer sedation durations than that with standard endoscopic procedures, as it involves navigating to the papilla or anastomosis of the HJ/PJ. Therefore, the appropriate selection and meticulous management of sedatives are essential during DB-ERCP.

Commonly used sedatives for endoscopic procedures include benzodiazepines, propofol, and dexmedetomidine [3]. Although propofol is increasingly being used, its application remains restricted in certain countries owing to safety concerns and the requirement for anesthesiologist supervision [3]. Benzodiazepines such as midazolam and diazepam are still considered standard sedatives for endoscopic procedures globally, including in the aforementioned countries [4,5]. Each member of the benzodiazepine family exhibits unique pharmacological properties. For example, diazepam has a half-life nearly 10 times longer than midazolam and cannot be diluted for small incremental dosing, increasing the potential risk of oversedation [3,6,7,8].

To date, no previous studies have compared various benzodiazepines for sedation in BAE, including DB-ERCP. This study evaluated the safety and efficacy of sedation in DB-ERCP by comparing outcomes between patients sedated with midazolam and those with diazepam.

## 2. Materials and Methods

### 2.1. Patients

**Figure 1:**
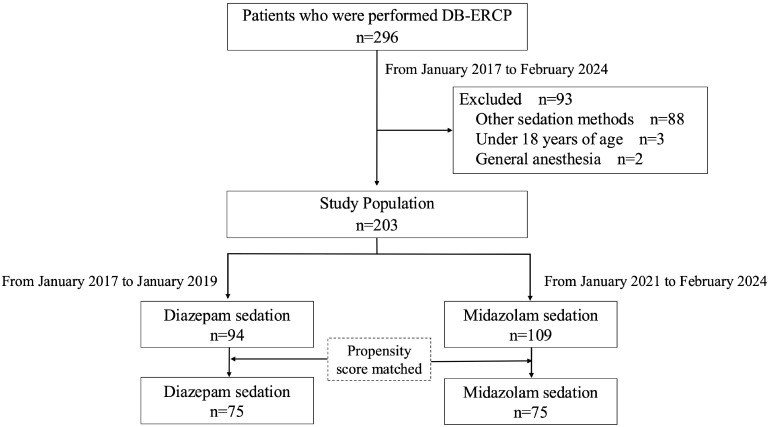
Schematic of the study design. DB-ERCP: double balloon–endoscopic retrograde cholangiopancreatography.

This retrospective study included 296 consecutively admitted patients who underwent DB-ERCP at Okayama University Hospital between January 2017 and February 2024 (Figure 1). Inclusion criteria were (1) patients with altered anatomy (Child method, pylorus-preserving pancreaticoduodenectomy-ⅡA and subtotal stomach-preserving pancreaticoduodenectomy-ⅡA, Roux-en-Y, or Billroth-II) and (2) patients requiring examination of a bile duct or pancreatic duct. Exclusion criteria were (1) patients under 18 years of age, (2) use of sedatives other than midazolam and diazepam, (3) general anesthesia, (4) pre-existing conditions such as hypotension (systolic blood pressure < 90 mmHg), bradycardia (heart rate < 50 beats/min), hypoxemia (oxygen saturation [SpO_2_] < 90%) or the need for oxygen supplementation before sedation, and (5) American Society of Anesthesiologists (ASA) physical status class Ⅳ or higher. Only the first procedure was analyzed for patients who underwent multiple DB-ERCP during the study period. A total of 203 patients met the inclusion criteria; 94 received diazepam sedation and 109 patients received midazolam sedation (Figure 1). Data were collected from electronic patient records. All patients provided written informed consent for treatment. This study was conducted in accordance with the principles of the Declaration of Helsinki and was approved by the Institutional Review Board of Okayama University Hospital (Approval Number: 2307-028).

### 2.2. Sedation Protocol and Monitoring

At our institution, diazepam was administered for sedation from January 2017 to January 2019, midazolam from January 2021 to February 2024, and propofol from February 2019 to December 2020. Midazolam and diazepam dosages were determined based on previous studies [9,10,11]. For diazepam (Teva Takeda, Nagoya, Japan) sedation, the initial loading dose was 5.0 mg for patients < 75 years old and 2.5 mg for those aged 75 years or older. After an intravenous loading dose of 2.5–5.0 mg diazepam and 17.5 mg pethidine, additional doses of diazepam (5.0 mg for patients < 75 years and 2.5 mg for those aged 75 years or older) or 17.5 mg pethidine were administered intravenously to maintain the required level of sedation. For midazolam-based sedation, the initial loading dose of midazolam (Sandoz Pharma K.K., Tokyo, Japan) was 2.0 mg for all patients. Following an intravenous loading dose of 2.0 mg midazolam and 17.5 mg pethidine (Takeda, Tokyo, Japan), additional doses of midazolam (2.0 mg for patients < 75 years and 1.0 mg for those aged 75 years or older) or 17.5 mg pethidine were administered intravenously to maintain the required level of sedation (Supplemental Table S1). The maximum doses of midazolam and diazepam were 10 and 20 mg, respectively.

Although various methods exist for assessing sedation depth, the Ramsay Sedation Scale (RSS) [12], recommended by the European Society of Gastrointestinal Endoscopy (ESGE) [5] (Supplemental Table S2), was employed in this study. Sedation levels were assessed at 5 min intervals from the start to the end of sedation. Assessments were conducted by both a physician and a nurse, and in cases of differing opinions, the higher score, reflecting deeper sedation, was recorded. The evaluations using RSS were performed by a trained physician who had completed in-hospital sedation training. In both groups, the target sedation level was 5–6 on the RSS, indicating deep sedation. If sedation levels fell below an RSS score of 5, additional bolus doses of the same sedative agent were administered at intervals of at least 3 min. If adequate sedation was not achieved even at the maximum dose, or if the sedation physician deemed further use of the same sedative insufficient because of disinhibition, the case was classified as “poor sedation requiring other sedatives,” and an additional sedative of a different type was administered. The procedure was discontinued in patients with severe respiratory depression or circulatory insufficiency. If the Aldrete score was ≤ 8 at the end of the DB-ERCP, a sedative antagonist (flumazenil; Terumo, Tokyo, Japan) or analgesic antagonist (naloxone; Alfresa Pharma Corporation, Osaka, Japan), or both, were administered as necessary [13].

Throughout the procedure, all patients were continuously monitored for heart rate, SpO_2_, and electrocardiographic changes using a bedside monitor (BSM-2301; Nihon Kohden Wellness Corporation, Tokyo, Japan). Blood pressure was measured automatically at 5 min intervals. All patients received supplemental oxygen at a flow rate of 2 L/min through a nasal cannula and were maintained in the prone posture during sedation. The procedures were conducted using either the EI-530B or EI-580BT double-balloon endoscopes (Fujifilm, Tokyo, Japan), with CO_2_ insufflation. Sedation was administered by a gastroenterologist who was not directly involved in performing the endoscopic procedures.

### 2.3. Outcomes and Definitions

The primary outcome was a comparison of the incidence of sedation-related adverse events (AEs), which included hypoxemia, bradycardia, and hypotension. These were defined as follows: hypoxemia, SpO_2_ < 90%; bradycardia, heart rate < 50 beats/min; hypotension, systolic blood pressure < 90 mmHg or a reduction of >20% [14,15]. Secondary outcomes included inadequate sedation requiring additional sedatives, sedation duration, dosage of infusion drugs, use of sedatives or analgesic antagonists, management and duration of sedation-related AEs, and identification of risk factors for sedation-related AEs.

### 2.4. Statistical Analysis

Propensity score matching was employed to identify matched cohorts within the two patient groups, minimizing confounding biases. The following covariates were included: age; sex; body mass index (BMI); current or ex-smoker status; alcohol abuse; narcotic/sedative use; underlying diseases, including cardiovascular or pulmonary disease, liver cirrhosis, and chronic renal failure; and ASA physical status, and type of intestinal reconstruction. The propensity scores were calculated using logistic regression models based on these covariates. One-to-one nearest neighbor matching without replacement was employed, with a caliper width of 0.2 to ensure proper balance between groups. Categorical variables are expressed as percentages, while continuous variables were reported as medians with interquartile ranges. Wilcoxon’s rank sum test and the Kruskal–Wallis test were used to compare continuous data. Fisher’s exact test was used to compare categorical data. Factors associated with sedation-related AEs were assessed using multivariate logistic regression analysis. Variables with a *p*-value < 0.05 in univariate analyses were included in the multiple logistic regression model, and odds ratios (OR) with 95% confidence intervals (CI) were calculated. Differences were considered statistically significant at *p* < 0.05. Data analysis was performed using the JMP Pro version 15 software for Mac (SAS Institute Inc., Cary, NC, USA).

## 3. Results

### 3.1. Patient Characteristics

A total of 203 patients met the eligibility criteria for this study, with 94 receiving diazepam and 109 receiving midazolam sedation. Following one-to-one propensity score matching, 75 patients from each group were included in the final analysis. Although a significant difference in the proportion of current or former smokers was initially observed between the two groups, this imbalance was resolved after matching, resulting in well-balanced patient distribution characteristics (Table 1).

### 3.2. Details of Sedation-Related Outcomes

Table 2 presents the sedation-related parameters of the two patient groups after propensity score matching. No significant differences were observed between the two groups in terms of induction time (*p* = 0.22), procedure time (*p* = 0.19), or total sedation time (*p* = 0.19). In the diazepam and midazolam sedation groups, induction doses of 5 mg (5–5 mg) and 3 mg (2–3 mg) and total doses of 10 mg (5–10 mg) and 4 mg (3–5 mg) were administered. Pethidine doses were comparable in both groups, with no significant differences observed in either the induction (*p* = 0.37) or total doses (*p* = 0.26). Similarly, there was no significant difference in the frequency of antagonist use for sedatives or analgesics between the groups (*p* = 0.85). Poor sedation requiring other sedatives was significantly more common in the diazepam sedation group compared with the midazolam patient group (8% [6/75] vs. 0% [0/75], respectively; *p* = 0.012). All six patients in the diazepam sedation group exhibiting poor sedation were successfully managed using propofol (1% Diprivan injection-kit; AstraZeneca, Osaka, Japan).

### 3.3. Sedation-Related AEs

Sedation-related AEs occurred more frequently in the diazepam group (28% [21/75]) than in the midazolam group (14% [11/75], *p* = 0.046) (Table 3). Among the various types of AEs, hypoxia was more common with diazepam sedation (19% [14/75]) than with midazolam (5% [4/75], *p* = 0.012). These patients were primarily managed by increasing oxygen flow (15/18, 83%) and performing jaw lifts (13/18, 72%), while one patient (1/18, 6%) in the diazepam sedation group required mask ventilation. In all cases of hypoxemia, the condition improved with the intervention, and no patient required intubation nor did any experience cardiopulmonary arrest. Hypotension incidence showed no significant difference between the two groups (diazepam sedation: 12% [9/75] vs. midazolam sedation: 8% [6/75], *p* = 0.41). Among the 15 patients with hypotension, only one (1/15, 7%) required vasopressor treatment, while the rest (14/15, 93%) improved with fluid infusion alone. All cases of hypotension improved following the intervention. Bradycardia was equally rare in both groups (diazepam sedation: 3% [2/75] vs. midazolam sedation: 3% [2/75], *p* = 1.0), and no patient required atropine sulfate treatment. An evaluation of the timing of sedation-related AEs revealed that hypoxemia occurred during the maintenance phase of sedation in all patients within the midazolam group. In contrast, five patients in the diazepam group experienced AEs following the endoscopy procedure. However, no procedures were discontinued because of AEs.

### 3.4. Risk Factors for Sedation-Related Adverse Events

Table 4 presents the results of the univariate and multivariate analyses examining risk factors associated with sedation-related AEs. In the univariate analysis, significant factors included BMI (OR, 3.5; 95% CI, 0.98–12; *p* = 0.043) and diazepam sedation (OR, 2.3; 95% CI, 1.0–5.1; *p* = 0.046). Multivariate analysis identified diazepam sedation as the sole risk factor for sedation-related AEs (OR, 2.3; 95% CI, 1.0–5.3; *p* = 0.048). Further analysis of the diazepam group revealed that both univariate and multivariate analyses identified male sex (male) (OR, 3.9; 95% CI, 1.2–16; *p* = 0.024) and high BMI (OR, 6.7; 95% CI, 1.1–57; *p* = 0.039) as significant risk factors for sedation-related complications (Supplemental Table S3).

## 4. Discussions

To our knowledge, this is the first study to compare the safety and efficacy of diazepam and midazolam for sedation during DB-ERCP. Sedation-related AEs were significantly more common with diazepam than with midazolam. Multivariate analysis identified diazepam sedation as an independent risk factor for sedation-related AEs. Additionally, poor sedation that led to the use of other sedative agents was significantly more common in diazepam sedation than midazolam sedation. These findings suggest that midazolam is safer and more effective than diazepam for DB-ERCP sedation.

Each member of the benzodiazepine family has distinct pharmacological properties. Midazolam offers advantages over diazepam owing to its shorter half-life and ability to be diluted for administration in smaller doses [3]. No prior studies have directly compared various benzodiazepines in the context of sedation for endoscopic treatments, including DB-ERCP. Consequently, the optimal benzodiazepine for endoscopic procedures remains undetermined. For upper gastrointestinal endoscopy, randomized controlled trials have shown that, compared to diazepam, midazolam provides superior outcomes such as greater patient satisfaction, enhanced amnesic effects regarding the examination, reduced phlebitis, and alleviation of patient discomfort [8,12,16]. A study comparing diazepam- and propofol-based sedation for DB-ERCP found a higher incidence of hypoxemia associated with diazepam [14]. The authors attributed this to challenges in precisely adjusting the bolus dose of diazepam and its longer half-life compared to other sedatives. In the present study, post-procedural hypoxemia occurred in five cases (7%) with diazepam sedation, whereas no cases (0%) were reported with midazolam. These findings indicate that the longer half-life and prolonged effects of diazepam contribute to postprocedural hypoxemia. We also observed that the occurrence of poor sedation leading to the use of other sedative agents was less frequent in the midazolam than the diazepam sedation groups (*p* = 0.012). Poor sedation occurred in six (8%) patients with diazepam sedation, and all of them were able to complete the endoscopy procedure with additional propofol.

A BMI > 25 kg/m^2^ was identified as a significant contributing factor for sedation-related AEs in the diazepam group. Previous reports have suggested that high BMI is a cause of hypoxemia and oversedation [17,18]. In obese patients, hypoxemia during sedation has been attributed to factors such as tongue base collapse, reduced thoracic mobility due to abdominal fat, excessive oxygen consumption from a high basal metabolic rate, and underlying obstructive sleep apnea. In patients with elevated BMI, meticulous sedation management and, if required, the preparation of supportive devices such as a pharyngeal airway are deemed crucial. For obese patients, adhering to a low-dose sedation protocol is essential, regardless of their higher body weight. Furthermore, the use of a CO₂ monitor is crucial for the early detection of ventilation impairments.

Several studies have compared the use of benzodiazepines with other sedatives for endoscopic procedures. For example, dexmedetomidine has been shown to enhance both patient and endoscopist satisfaction during ERCP, although it is associated with lower heart rates during the procedure [19]. This sedative is particularly useful for maintaining prolonged sedation without causing respiratory depression; however, its broader adoption has been limited by certain drawbacks, including a complex fixed initial loading method, the risk of hemodynamic instability when used as a monotherapy, and its higher cost relative to other sedatives [20,21,22,23].

Propofol, another widely used sedative, offers advantages such as a rapid onset, high recovery quality, and minimal procedural interruptions [24,25]. Previous studies have shown that propofol sedation is associated with lower incidence rates of inadequate sedation, vigorous body movements, and hypoxemia compared to diazepam during DB-ERCP [14]. However, the lack of specific reversal agents for propofol (unlike benzodiazepines) is disadvantageous. Furthermore, owing to its narrow therapeutic window, the safe administration of propofol by non-anesthesiologists necessitates the use of supportive devices such as target-controlled infusion (TCI) systems and capnography [26,27]. Although the safety of propofol administration by trained non-anesthesiologists has been well documented [28,29], its routine use remains challenging in countries where anesthesiologist supervision is often required. In many regions, the lack of educational systems and guidelines for non-anesthesiologists to safely administer propofol is a concern. Consequently, considering the background of other sedatives discussed, sedation with benzodiazepines and opioids is still considered the standard approach for endoscopic treatment globally [4,5]. Therefore, further research is necessary to establish the optimal choice of benzodiazepines for endoscopic treatment.

The limitations of this study were as follows: First, this was a retrospective, single-center study with a relatively small sample size, which may have introduced a selection bias. Therefore, the results were analyzed using propensity score matching to minimize this bias. Second, variations in the endoscopic equipment used over time may have influenced the overall duration of the procedure. Third, this study did not compare the effects of propofol as a sedative, despite its widespread use as an anesthetic for balloon endoscopy in Europe and the United States. Fourth, the sedative dosages used in the study were standardized and not tailored to individual patients, which could have resulted in significant variability in patient responses to the sedatives. Fifth, the quality of sedation assessment depends on the training level of sedation physicians. Internal qualification standards have been established at our institution, but a broader and more standardized system for training and evaluating sedation physicians involved in sedation management is needed.

In conclusion, midazolam offers a safer and more effective sedation alternative to diazepam for DB-ERCP. However, further large-scale prospective studies are warranted to validate these findings and provide guidance for clinical practice.

## Figures and Tables

**Table 1:** Clinical characteristics of the patients in the present study.

	Unmatched			Propensity Score Matched		
Parameters	Diazepam Sedation (*n* = 94)	Midazolam Sedation (*n* = 109)	*p* Value	Diazepam Sedation (*n* = 75)	Midazolam Sedation (*n* = 75)	*p* Value
Age, median (IQR), years	73 (66–78)	72 (63–77)	0.29	73 (66–77)	71 (63–78)	0.57
Sex, male/female	63/31	72/37	0.88	46/29	49/26	0.61
BMI, median (IQR), kg/m^2^	21 (18–23)	20 (19–22)	0.64	20 (19–22)	20 (18–22)	0.99
Current or ex-smoker, *n* (%)	37 (39)	28 (26)	0.037	22 (29)	21 (28)	0.86
Alcohol abuse, *n* (%)	13 (14)	13 (12)	0.69	8 (11)	8 (11)	1.0
Narcotic/sedative use, *n* (%)	20 (21)	22 (20)	0.85	16 (21)	14 (19)	0.68
Underlying disease, *n* (%)						
Cardiovascular disease	13 (14)	16 (15)	0.86	8 (11)	5 (7)	0.38
Pulmonary disease	11 (12)	10 (9)	0.56	8 (11)	7 (9)	0.79
Liver cirrhosis	7 (7)	9 (8)	0.83	6 (8)	4 (5)	0.51
Chronic renal failure	4 (4)	5 (5)	0.91	4 (5)	3 (4)	0.70
ASA-PS, *n* (%) *			0.47			0.63
1	52 (55)	53 (49)		38 (51)	42 (56)	
2	38 (40)	53 (49)		33 (44)	31 (41)	
3	4 (4)	3 (3)		4 (5)	2 (3)	
Type of intestinal reconstruction, *n* (%)			0.62			0.70
Child	51 (54)	64 (59)		41 (55)	46 (61)	
Roux-en-Y	37 (39)	41 (38)		30 (40)	26 (35)	
Billroth-II	6 (6)	4 (4)		4 (5)	3 (4)	

IQR, interquartile range; BMI, body mass index; ASA-PS, American Society of Anesthesiologists physical status. * ASA-PS: 1: A normal healthy patient; 2: A patient with mild systemic disease; 3: A patient with severe systemic disease, not incapacitating.

**Table 2:** Sedation efficacy measurements and infusion drug doses.

	Diazepam Sedation (*n* = 75)	Midazolam Sedation (*n* = 75)	*p* Value
Induction time, median (IQR), min	3 (2–5)	4 (3–5)	0.22
Procedure time, median (IQR), min	56 (35–80)	50 (32–69)	0.19
Total sedation time, median (IQR), min	58 (38–82)	54 (36–71)	0.19
Induction diazepam/midazolam dose, median (IQR), mg	5 (5–5)	3 (2–3)	-
Total diazepam/midazolam dose, median (IQR), mg	10 (5–10)	4 (3–5)	-
Induction pethidine dose, median (IQR), mg	35 (18–53)	35 (18–35)	0.37
Total pethidine dose, median (IQR), mg	53 (35–70)	53 (35–70)	0.26
Use of antagonist for sedative and/or analgesic, *n* (%)	19 (25)	20 (27)	0.85
Poor sedation requiring other sedative agents	6 (8)	0 (0)	0.012

IQR, interquartile range.

**Table 3:** Sedation-related adverse events and their management.

	Diazepam Sedation (*n* = 75)	Midazolam Sedation (*n* = 75)	*p* Value
All adverse events	21 (28)	11 (14)	0.046
Hypoxemia, *n* (%)	14 (19)	4 (5)	0.012
Period of hypoxemia			0.036
Induction period	2 (3)	0 (0)	
Maintenance period	7 (9)	4 (5)	
After procedure	5 (7)	0 (0)	
Hypotension, *n* (%)	9 (12)	6 (8)	0.41
Period of hypotension			1.0
Induction period	0 (0)	0 (0)	
Maintenance period	9 (12)	5 (7)	
After procedure	0 (0)	1 (1)	
Bradycardia, *n* (%)	2 (3)	2 (3)	1.0
Period of bradycardia			1.0
Induction period	0 (0)	0 (0)	
Maintenance period	2(3)	2(3)	
After procedure	0 (0)	0 (0)	
Discontinuance of procedure owing to adverse event, *n* (%)	0 (0)	0 (0)	NA

NA, not available.

**Table 4:** Univariate and multivariate analysis for predictive factors affecting adverse events.

	Adverse Event (*n* = 32)	No Adverse Event (*n* = 118)	Univariate Analysis			Multivariate Analysis		
			OR	95%CI	*p* Value	OR	95%CI	*p* Value
Age, *n* (%)					0.92			
>75 years	10 (21)	38 (79)	0.96	0.41–2.2				
≤75 years	22 (22)	80 (78)	1					
Sex, *n* (%)					0.050			
Male	25 (26)	70 (74)	2.4	0.98–6.1				
Female	7 (13)	48 (87)	1					
BMI, *n* (%)					0.043			0.066
>25 kg/m^2^	5 (45)	6 (55)	3.5	0.98–12		3.5	0.92–13	
≤25 kg/m^2^	27 (19)	112 (81)	1			1		
ASA-PS, *n* (%)					0.71			
≥Class 2	14 (20)	56 (80)	0.86	0.39–1.9				
Class 1	18 (23)	62 (78)	1					
Alcohol abuse, *n* (%)					0.12			
Yes	1 (6)	15 (94)	0.22	0.028–1.7				
No	31 (23)	103 (77)	1					
Smoking history, *n* (%)					0.72			
Yes	10 (23)	33 (77)	1.2	0.50–2.7				
No	22 (21)	85 (79)	1					
Narcotic/sedative use, *n* (%)					0.84			
Yes	6 (20)	24 (80)	0.90	0.33–2.4				
No	26 (22)	94 (78)	1					
Underlying disease, *n* (%)								
Cardiovascular disease					0.21			
Yes	1 (8)	12 (92)	0.28	0.036–2.3				
No	31 (23)	106 (77)	1					
Pulmonary disease					0.60			
Yes	4 (27)	11 (73)	1.4	0.41–4.7				
No	28 (21)	107 (79)	1					
Liver cirrhosis					0.37			
Yes	1 (10)	9 (90)	0.39	0.048–3.2				
No	31 (22)	109 (78)	1					
Chronic renal failure					0.63			
Yes	2 (29)	5 (71)	1.5	0.28–8.1				
No	30 (21)	113 (79)	1					
Indication for DB-ERCP, *n* (%)					0.94			
Hepaticojejunostomy anastomotic stricture	19 (21)	71 (79)	0.97	0.44–2.1				
Others	13 (22)	47 (78)	1					
Type of intestinal reconstruction, *n* (%)					0.66			
Roux-en-Y	13 (23)	43 (77)	1.2	0.54–2.7				
Others	19 (20)	75 (80)	1					
Total sedation time, *n* (%)					0.19			
>60 min	18 (26)	51 (74)	1.7	0.77–3.7				
≤60 min	14 (17)	67 (83)	1					
Diazepam sedation, *n* (%)					0.046			0.048
Yes	21 (28)	54 (72)	2.3	1.0–5.1		2.3	1.0–5.3	
No	11 (15)	64 (85)	1			1		
Total pethidine dose, *n* (%)					0.64			
>70 mg	10 (24)	32 (76)	1.2	0.52–2.9				
≤70 mg	22 (20)	86 (80)	1					

OR, odds ratio: CI, confidence intervals: BMI, body mass index; ASA-PS, American Society of Anesthesiologists physical status: DB-ERCP, double balloon–endoscopic retrograde cholangiopancreatography.

## Data Availability

The datasets used in the current study can be obtained from the corresponding author upon reasonable request.

## Supplementary Materials

The following supporting information can be downloaded at https://www.mdpi.com/article/10.3390/jcm14072287/s1: Supplemental Table S1: The dose of sedatives and analgesics for patients undergoing DB-ERCP; Supplemental Table S2: Ramsey sedation scale; Supplemental Table S3: Univariate and multivariate analysis for predictive factors of adverse event in diazepam group.
