# Supplementary material for: Comparison of Midazolam and Diazepam for Sedation in Patients Undergoing Double-Balloon Endoscopic Retrograde Cholangiopancreatography: A Propensity Score-Matched Analysis
Source: J Clin Med. 2025 Mar 27;14(7):2287. doi: 10.3390/jcm14072287 (PMC11989694; doi:10.3390/jcm14072287)
Supplement: Supplementary file 1 [file jcm-14-02287-s001.zip › jcm-3527742-supplementary.pdf]

Supplementary Material

Author Names

Yuki Fujii, Kazuyuki Matsumoto, Kei Harada, Nao Hattori, Ryosuke Sato, Taisuke Obata, Akihiro Matsumi, Kazuya Miyamoto, Daisuke Uchida, Shigeru Horiguchi, Koichiro Tsutsumi, Toshiharu Mitsuhashi, Motoyuki Otsuka

Title of Paper

Comparison of midazolam and diazepam for sedation in patients undergoing double-balloon endoscopic retrograde cholangiopancreatography: A propensity score-matched analysis

Supplemental table S1. The dose of sedatives and analgesics for patients undergoing DB-ERCP

	Diazepam sedation		Midazolam sedation	
	< 75 years old	≥ 75 years old	< 75 years old	≥ 75 years old
Loading dose	Diazepam 5mg and Pethidine 17.5mg	Diazepam 2.5mg and Pethidine 17.5mg	Midazolam 2mg and Pethidine 17.5mg	Midazolam 2mg and Pethidine 17.5mg
Bolus dose	Diazepam 5mg or Pethidine 17.5mg	Diazepam 2.5mg or Pethidine 17.5mg	Midazolam 2mg or Pethidine 17.5mg	Midazolam 1mg or Pethidine 17.5mg

*Repetitive dosing to keep the RSS level at 5-6 was possible. Bolus interval was at least 3 minutes.

DB-ERCP, double balloon-enteroscopic retrograde cholangiopancreatography: RSS, ramsay sedation scale

Supplemental table S2. Ramsey sedation scale

Score	Response
1	Anxious, agitated, restless
2	Cooperative, oriented, tranquil
3	Responsive to commands only
4	Brisk response to light glabellar tap or loud auditory stimulus
5	Sluggish response to light glabellar tap or loud auditory stimulus
6	No response to light glabellar tap or loud auditory stimulus

Supplemental table S3. Univariate and multivariate analysis for predictive factors of adverse event in diazepam group

	Adverse event (n=21)	No adverse event (n=54)	Univariate analysis			Multivariate analysis		
			OR	95%CI	P value	OR	95%CI	P value
Age, n (%)					0.75			
>75 years	7 (30)	16 (70)	1.2	0.40-3.5				
≦75 years	14 (27)	38 (73)	1					
Sex, n (%)					0.030			0.024
Male	17 (37)	29 (63)	3.7	1.1-12		3.9	1.2-16	
Female	4 (14)	25 (86)	1			1		
BMI, n (%)					0.028			0.039
>25 kg/m2	4 (67)	2 (33)	6.1	1.0-36		6.7	1.1-57	
≦25 kg/m2	17 (25)	52 (75)	1			1		
ASA-PS, n (%)					0.22			
≧Class2	8 (22)	29 (78)	0.53	0.19-1.5				
Class1	13 (34)	25 (66)	1					
Aclohol abuse, n (%)					0.30			
Yes	1 (13)	7 (88)	0.34	0.039-2.9				
No	20 (30)	47 (70)	1					
Smoking history, n (%)					0.64			
Yes	7 (32)	15 (68)	1.3	0.44-3.8				
No	14 (26)	39 (74)	1					
Narcotic/sedative use, n (%)					0.76			
Yes	4 (25)	12 (75)	0.82	0.233-2.9				
No	17 (29)	42 (71)	1					
Underlying disease, n (%)								
Cardiovascular disease					0.30			
Yes	1 (13)	7 (88)	0.34	0.039-2.9				
No	20 (30)	47 (70)	1					
Pulmonary disease					0.84			
Yes	2 (25)	6 (75)	0.84	0.16-4.5				
No	19 (28)	48 (72)	1					
Liver cirrhosis					0.11			
Yes	0 (0)	6 (100)	NA	NA				
No	21 (30)	48 (70)	1					
Chronic renal failure					0.31			
Yes	2 (50)	2 (50)	2.7	0.36-21				
No	19 (27)	52 (73)	1					
Indication for BAE-ERCP, n (%)					0.40			
Hepaticojejunostomy anastomotic stricture	11 (24)	34 (76)	0.65	0.23-1.8				
Others	10 (33)	20 (67)	1					

Type of intestinal reconstruction, n (%)					0.75
Roux-en-Y	9 (30)	21 (70)	1.2	0.43-3.3	
Others	12 (27)	33 (73)	1		
Total sedation time, n (%)					0.64
>60min	11 (31)	25 (69)	1.3	0.46-3.5	
≦60min	10 (26)	29 (74)	1		
Total pethidine dose, n (%)					0.38
>70mg	7 (23)	24 (77)	0.63	0.22-1.8	
≦70mg	14 (32)	30 (68)	1		

OR, odds ratio: CI, confidence intervals: BMI, body mass index; ASA-PS, American Society of Anesthesiologists performance status, BAE-ERCP, balloon assisted enteroscopy-guided endoscopic retrograde cholangiopancreatography
